# Hippocalcin signaling via site-specific translocation in hippocampal neurons

**DOI:** 10.1016/j.neulet.2008.06.089

**Published:** 2008-09-12

**Authors:** Olga Markova, Daniel Fitzgerald, Andrey Stepanyuk, Alexandr Dovgan, Volodymyr Cherkas, Alexei Tepikin, Robert D. Burgoyne, Pavel Belan

**Affiliations:** a Bogomoletz Institute of Physiology, Kiev 01024, Ukraine; b Physiological Laboratory, School of Biomedical Sciences, University of Liverpool, Liverpool L69 3BX, UK

**Keywords:** Signal transduction, NCS proteins, Ca^2+^, Cellular response, Hippocampal neurons

## Abstract

Hippocalcin is a Ca^2+^-binding protein, which belongs to the family of neuronal Ca^2+^ sensors. It is highly expressed in the hippocampus but molecular mechanisms underlying its action in this part of the brain have not been investigated in detail. To study whether intrinsic neuronal activity could result in hippocalcin-mediated signal transduction we examined spontaneous and action potential (AP)-dependent changes in fluorescence of yellow fluorescent protein-tagged hippocalcin (HPCA-YFP) in transiently transfected hippocampal cultured neurons. In 6–12 DIV neurons HPCA-YFP spontaneously translocated longitudinally to specific sites within diffusionally confined domains of neuronal processes. The translocations to these sites were expressed as fast, reversible increases in HPCA-YFP fluorescence coincided with a decrease in adjacent sites indicating genuine protein translocation. Physiologically relevant neuronal stimulation with short trains of action potentials also resulted in fast, simultaneous, reversible, and [Ca^2+^]_i_-dependent translocations of HPCA-YFP to several sites synchronizing hippocalcin signaling in different parts of neuronal processes. The amount of translocated protein increased with the number of action potentials in a train decoding the number of APs into the amount of translocated protein. We conclude that hippocalcin may signal within diffusionally restricted domains of neuronal processes in which it might play a physiological role in Ca^2+^-dependent local activation of specific molecular targets.

A number of neuronal Ca^2+^ sensor (NCS) proteins are expressed in the nervous system, where they have distinct roles in regulation of cell functions [3]. A member of this family, hippocalcin, is specifically highly expressed in the hippocampal neurons; in particular in their dendrites and axons [17], suggesting that it might be involved in pre- and post-synaptic signaling. It has been shown that Ca^2+^-dependent hippocalcin activation in these neurons is one of the necessary steps involved in expression of NMDA receptor dependent long-term depression (LTD) [17] and in production of a slow afterhyperpolarization (sAHP) [19].

The molecular mechanism, by which hippocalcin operates, is thought to be a Ca^2+^/myristoyl switch [3]. Hippocalcin has a lipophilic myristoyl group sequestered in the Ca^2+^ free form of the protein. Following Ca^2+^-binding a substantial conformational change allows extrusion of the lipophilic group [1] that may result in the protein translocation from cytosol to membranes. It is obvious that neurons might use this property of hippocalcin in signal transduction processes [13]. The endogenous, closely related NCS proteins, VILIP-1 and VILIP-3, can also translocate to membranes [18]. However, the existence and spatio-temporal dynamics of translocation of NCS proteins driven by internal neuronal processes have not yet been investigated in its native environment.

Hippocampal neurons within neuronal networks reveal network-driven activity leading to a complex spatio-temporal patterns of free Ca^2+^ concentration ([Ca^2+^]_i_) changes in their processes [2]. We hypothesized that these changes could be decoded by hippocalcin via its translocation to membranous sites containing its specific targets. To validate this hypothesis we have examined spontaneous and action potential-induced changes in fluorescence of hippocalcin tagged by yellow fluorescent protein (HPCA-YFP) in hippocampal neurons growing in low-density cultures.

All procedures used in this study were approved by the Animal Care Committee of Bogomoletz Institute of Physiology and conform to the Guidelines of the National Institutes of Health on the Care and Use of Animals. Neurons were obtained from newborn rats (postnatal day 0–1) killed via rapid decapitation without anesthesia. Hippocampi of the rats were enzymatically dissociated with trypsin. The cells were plated on glass coverslips coated with laminin and poly-l-ornithine (Invitrogen, USA) and maintained in feeding solution consisted of minimal essential medium, 10% horse serum and other necessary and previously described additives (Invitrogen, USA) in humidified atmosphere containing 5% CO_2_ at 37 °C [9].

Hippocampal neurons were transfected after 5–9 days in culture using the DNA–calcium phosphate precipitation method essentially as described by a supplier (ProFection Mammalian Transfection System, Promega). All cultures were used for the experiments at 2–5 days after transfection. Transfection efficiency varied from 5% to 30% depending on DIV.

Neurons growing in the cultures were visualized using an inverted microscope (IX70, Olympus, Germany). Whole-cell patch-clamp recordings were obtained from neurons using an EPC-10/2 amplifier (HEKA, Germany). The composition of extracellular solution was as follows (mM): NaCl 140; KCl 2; CaCl_2_ 2; MgCl_2_ 1; HEPES 10; glucose 10; pH 7.3; osmolarity 300 mOsm. An intracellular solution contained (mM): KCl 140; NaCl 5; CaCl_2_ 0.1–0.3; EGTA 1; MgATP 2; GTP 0.3; HEPES 10; pH 7.3; osmolarity 290 mOsm. Patch electrodes were pulled to a resistance of 3–5 MΩ. Membrane voltage or transmembrane current were low-pass filtered (3 kHz) and acquired at 10 kHz.

In some experiments, neurons were extracellularly stimulated via a patch pipette using a stimulator isolator (ISO-Flex, A.M.P.I., Israel). The stimulation pipette was filled with an extracellular solution and voltage pulses (0.5–2.0 ms; 20–40 V) were applied at the frequency of 10–30 Hz. All experiments were conducted at room temperature.

Time-lapse video imaging of transiently transfected hippocampal neurons was performed using TILL Photonics imaging system (TILL Photonics, Germany). A customized routine written in TILLvision software was used to calculate relative changes in HPCA-YFP fluorescence against initial background or against CFP fluorescence in order to determine sites of HPCA-YFP fluorescence changes. A value of translocation, Δ*F*/*F*, was expressed as relative changes in HPCA-YFP fluorescence.

Spontaneous activity observed in both the hippocampus and primary hippocampal cultures during early postnatal development should result in fast (rising time less than a second) elevation of [Ca^2+^]_i_ [2]. Based on our previous results showing that hippocalcin can translocate in less than a second [13] we specifically searched for spontaneous localized elevations in HPCA-YFP fluorescence occurring in a time window of 0.5–2.0 s.

Hippocampal neurons were co-transfected with plasmids carrying genes of HPCA-YFP [12] and cyan fluorescent protein, CFP. The latter protein was used as a control for any morphological re-arrangements. HPCA-YFP was diffusely distributed throughout soma, dendrites and axons of hippocampal neurons (Fig. 1A). Fig. 1B represents an example of fast spontaneous elevations in HPCA-YFP fluorescence observed in several local sites within the dendritic tree of a cultured hippocampal neuron. Three types of events were well distinguished in the recordings: (i) short (3–30 s) one peak transient, (ii) multiple peak transients (Fig. 1Ba), and (iii) long (1–3 min) plateau (not shown). The first type of events made up more than 90% of observed HPCA-YFP fluorescence increases and further statistical analysis will be restricted to this type of events.

The HPCA-YFP fluorescence transients (76 transients recorded in 19 neurons) had a rising time of 1–5 s and a decay time of 5–20 s (mean duration at a half height 20 ± 12 s). They repeatedly occurred often in the same places within dendritic trees and axons and each of them was spatially restricted to the area of 1–5 μm (mean value 3.8 ± 2.2 μm). Transient amplitudes were in a range from several up to 100% of initial level of fluorescence (mean value 30 ± 20%). In all cases of these fast transient elevations HPCA-YFP fluorescence was completely reversed to its initial value. No changes in CFP fluorescence were observed in the same sites indicating a genuine increase in HPCA-YFP fluorescence rather than morphological re-arrangements (26.5 ± 4.0% and 0.9 ± 2.0% for amplitudes of HPCA-YFP and CFP fluorescence changes in the same regions of interest (ROIs) at the maximum of HPCA-YFP changes; *p* < 0.01; 43 events recorded in 8 neurons).

The binding of calcium ions and the Ca^2+^/myristoyl switch could in theory lead to conformational change in the YFP part of the chimeric molecule and consequently to changes of YFP fluorescence. Therefore, the observed increase in HPCA-YFP fluorescence (Fig. 1) could be both due to changes of HPCA-YFP conformation and due to HPCA-YFP translocations. In order to discriminate between these possibilities the HPCA-YFP fluorescence was quantified in the vicinity of sites where the increase in HPCA-YFP fluorescence was recorded. Fig. 1C represents a fluorescence image of a hippocampal neuron (10 DIV; co-transfected with HPCA-YFP and CFP), in which an increase in HPCA-YFP fluorescence was observed in a distinct site in the neuronal dendritic tree marked by a rectangular box shown with a higher magnification in Fig. 1D and E. An image in this rectangular box with a maximal increase in HPCA-YFP fluorescence during a spontaneous event was subtracted from an average of five images taken right before initiation of the increase. A pseudocolor palette, in which pixel values exceeding two standard deviations of background noise were represented in yellow and those that were less by the same value were represented in white, was applied to this differential image (Fig. 1E). It is clearly seen that the site of about 5 μm with the increase in HPCA-YFP fluorescence is surrounded by two sites where the fluorescence was decreased. A time course of HPCA-YFP fluorescence was calculated in a set of ROIs placed over ‘yellow’ and ‘white’ areas and a ROI covering the whole area where any changes in HPCA-YFP fluorescence were observed (Fig. 1F). A robust increase in the ‘yellow’ site coincided with moderate but significant decrease in the neighboring ‘white’ sites indicating longitudinal rather than transverse translocation of HPCA-YFP. A total value of fluorescence in the ROI covering the whole area of fluorescence changes was practically constant during the spontaneous event (Fig. 1F). The total value of HPCA-YFP fluorescence in the whole area of fluorescence changes in other such ROIs (30 events recorded in 9 neurons) was not substantially changed during the translocations, indicating a genuine translocation of HPCA-YFP as the main reason of the observed spontaneous fluorescence changes. Most probably cytosolic HPCA-YFP molecules from regions immediately adjacent to translocation sites translocated to membranes locally decreasing cytosolic HPCA-YFP concentration. This led to HPCA-YFP diffusion from neighboring cytosolic regions and additional HPCA-YFP insertion in the translocation sites.

To validate that we also followed a spatio-temporal pattern of translocations (*n* = 9). For that HPCA-YFP fluorescence intensity along the dendrite (see a black curve in Fig. 1D) divided by respective CFP fluorescence intensity was plotted as a function of time (Fig. 1G). The translocation events covered a distinct part of the dendritic branch (about 15–30 μm) in which HPCA-YFP was initially translocated from regions immediately adjacent to the translocation site with more distal regions involved later on (Fig. 1G). The rate at which a decrease in HPCA-YFP fluorescence was propagated along the dendritic branch (several μm/s) is in line with cytosolic HPCA-YFP diffusion [13]. Thus, we have concluded that HPCA-YFP can spontaneously translocate to distinct membranous sites in dendrites and axons of hippocampal neurons and these translocations occur in diffusionally restricted domains of neuronal processes.

At the next step we studied if action potential-induced activation of voltage-operated calcium channels can lead to HPCA-YFP translocation. In order not to disturb an intracellular environment, intrinsic [Ca^2+^]_i_ regulation and HPCA-YFP spatial distribution extracellular stimulation of neurons was initially employed.

Fig. 2 demonstrates a representative example of experiments in which a neuron co-transfected with HPCA-YFP and CFP was extracellularly stimulated (20–30 times at 30 Hz) through an electrode located nearby neuronal somata. An extracellular solution in this series of experiments contained a cocktail of glutamate and GABA_A_ receptor blockers (DL-AP5, 50 μM; CNQX, 10 μM; bicuculline, 10 μM) in order to suppress spontaneous network activity and to have voltage-operated Ca^2+^ channels as the only source of Ca^2+^ influx. Simultaneous, synchronous and robust HPCA-YFP translocations to two closely situated but different sites were observed in a neuronal process as a result of the stimulation (Fig. 2A–C). As in the case of spontaneous translocations no change in CFP fluorescence was recorded in HPCA-YFP translocation sites. Increases in HPCA-YFP fluorescence in some sites coincided in these experiments with the fluorescence decreases in neighboring sites validating genuine HPCA-YFP translocations (Fig. 2A–C). In analogous experiments (45 translocations recorded in 5 neurons) extracellular stimulations induced HPCA-YFP translocation in different neuronal processes to sites of 1–5 μm of length (1.4 ± 0.8 μm). Recovery of the initial pattern of HPCA-YFP distribution occurred in 7–30 s (mean value 8.4 ± 3.2 s). A mean value of amplitudes of translocation transients was 13 ± 8% that was significantly lower than amplitudes of spontaneous translocation. This indicates that involvement of different sources of Ca^2+^ (voltage-operated Ca^2+^ channels in a case of APs vs. synaptically induced Ca^2+^ influx in a case of spontaneous network activity) can be differentially decoded by hippocalcin translocation. As in the case of spontaneous activity, extracellular stimulation induced HPCA-YFP translocation to a small area along the processes from the neighboring sites rather than its lateral translocation from a cytosol to the plasma membrane. Synchronous translocations to many sites were also routinely observed in practically all (92%) tested neurons.

Spontaneous and AP-dependent translocation occurred in the same way in a wide range of HPCA-YFP expression levels indicating that overexpression of the protein hardly affects its translocation.

Repeated extracellular stimulations, as well as APs induced in a current clamp mode, resulted in HPCA-YFP translocation to the same sites within the neuronal processes. The amount of translocated HPCA-YFP was equal in a response to equal consequently delivered stimulations and distribution of HPCA-YFP fluorescence was completely recovered after each stimulation episode; the amplitudes of first and fourth translocation transients were not significantly different (*p* > 0.2; Fig. 2D and E). Increasing the number of APs in the trains resulted in an increase in amplitudes of respective translocations in dendritic branches in a range up to 50 APs; longer stimulations resulted in some saturation of the translocation responses (Fig. 2F). HPCA-YFP was extremely sensitive to neuronal activity with translocations induced by a few APs in axons of hippocampal neurons being routinely observed (not shown). A spatial analysis of HPCA-YFP translocations in the same part of neuronal process in response to the trains with different numbers of APs showed that HPCA-YFP fluorescence increases in a site of translocation were always correlated with HPCA-YFP fluorescence decreases in the adjacent parts of the same process (Fig. 2B, C and F). That is, the stimulation strength was decoded in a fractional part of HPCA-YFP longitudinally translocated to a distinct site within a given part of process. Thus, the results of these experiments additionally support our initial observations that hippocalcin signals within diffusionally restricted domains of neuronal processes in which hippocalcin signaling occurs separately from other parts of the same neuronal processes. In these domains hippocalcin might decode trains of action potentials transforming their number into the amount of protein translocated to its targets.

The translocation of HPCA-YFP to membranes should depend on the binding of Ca^2+^ [13]. Therefore, we addressed the question of whether Ca^2+^ entry during depolarization of plasma membrane is necessary for observed HPCA-YFP translocation. Transfected hippocampal neurons were loaded with a fluorescent calcium dye fura-2 (0.1–0.2 mM) via a patch pipette allowing cytosolic Ca^2+^ concentration to be measured in parallel with HPCA-YFP translocation. We mainly used a perforated patch-clamp configuration with β-escin as a pore forming substance. Pores that are formed by β-escin are permeable for water-soluble molecules with a molecular weight up to 10 kDa [4] and are not permeable for larger molecules. Thus, with this experimental design we had a possibility to load cells with fura-2 and simultaneously preserve HPCA-YFP intracellular distribution.

Several episodes of strong stimulation in a voltage clamp mode (100 stimuli at 16 Hz; depolarization from −60 mV to 0 mV for 10 ms) were applied to neurons transfected with HPCA-YFP plasmid. These stimulations were performed in extracellular solutions with normal (2.0 mM) and low (0.5 mM) levels of Ca^2+^ concentrations. HPCA-YFP translocated in the normal extracellular solution while HPCA-YFP translocations were significantly decreased (to 14 ± 4% of control; *p* < 0.05; 32 ROIs recorded in 6 neurons; Fig. A Supplementary Results) when extracellular Ca^2+^ concentration was decreased. A wash-in of Ca^2+^ resulted in resumption of the translocations (83 ± 8% of control).

Ca^2+^ binding to EF-hand motifs of hippocalcin initiates Ca^2+^/myristoyl switch [13]. To prevent Ca^2+^ binding by EF-hands 2 and 3 we used a construct with glutamates at positions 85 and 121 mutated to glutamines, HPCA (E85, 121Q)-CFP [12]. Hippocampal neurons were co-transfected to express HPCA-YFP and HPCA (E85, 121Q)-CFP. When [Ca^2+^]_i_ was elevated by induction of APs HPCA-YFP translocations were routinely observed whereas HPCA (E85, 121Q)-CFP translocated neither to the sites where HPCA-YFP does (16.3 ± 3.2% and 1.3 ± 0.7% for a wild type and mutant, respectively; *p* < 0.01; Fig. A Supplementary Results) nor to any other sites. These results are consistent with a suggestion that Ca^2+^/myristoyl switch is required for a site-specific HPCA-YFP translocation observed in the experiments. Both types of experiments also demonstrate that HPCA-YFP translocates in hippocampal neurons in a Ca^2+^-dependent manner.

Thus, we have shown for the first time that neuronal calcium sensor proteins that posses a Ca^2+^–myristoyl switch mechanism can be subjected to fast, reversible, site-specific and Ca^2+^-dependent translocations as a result of intrinsic [Ca^2+^]_i_ dynamics. The translocation of the NCS protein, hippocalcin, has now been observed in hippocampal neurons—a natural cellular system in which hippocalcin is most prominently expressed endogenously in the rat CNS [6]. Hippocalcin translocated to many sites within processes of cultured hippocampal neurons. These translocations may occur synchronously and we have demonstrated that action potentials are a mechanism synchronizing hippocalcin translocations to different sites. It has been also shown that [Ca^2+^]_i_-dependent hippocalcin signaling occurs in diffusionally restricted domains of neuronal processes that may signal as independent units.

Hippocalcin has been suggested to specifically bind many proteins [5] and play many roles in the Ca^2+^-dependent signal transduction of physiological and pathological processes in the central nervous system [17,19,11,15,8,7,14,10,16]. Recent results have shown that calcium-dependent phosphorylation of cAMP-response element-binding protein, CREB, is significantly attenuated in hippocampal neurons of hippocalcin-deficient mice [7]. Thus, hippocalcin might be involved in the regulation of protein expression but it is unlikely to be related to fast and reversible hippocalcin translocations in neuronal processes observed in this study. Possibly hippocalcin may take part in different signaling mechanisms within different compartments of the hippocampal neurons. Even in the neuronal processes several different functions of hippocalcin could be potentially suggested based upon the qualitatively different spatio-temporal patterns of observed HPCA-YFP translocations.

It is interesting to note that hippocalcin-deficient mice were impaired on spatial and associative memory tasks [7] indicating hippocalcin involvement in synaptic plasticity. Recent studies have further validated this suggestion showing that hippocalcin may function as a calcium sensor in hippocampal long-term depression playing a crucial Ca^2+^-sensing role in NMDAR-dependent endocytosis of synaptic AMPA receptors [17]. It was suggested that hippocalcin, binds an adaptor protein, AP2, and translocates to the plasma membrane; followed by AP2 interaction with AMPARs and their clathrin-coated endocytosis. It is being known that interaction between hippocalcin and AP-2–AMPAR complex is transient and may only occur at the plasma membrane, which is in line with our results showing that hippocalcin translocations are transient, site-specific and completely reversible.

A recent work, showing that Ca^2+^ binding to hippocalcin is one of the necessary steps involved in the production of slow afterhyperpolarization (sAHP) [19], also supports the idea of numerous roles of hippocalcin in intracellular signaling. AP-induced [Ca^2+^]_i_ elevations employed in our studies are analogous to ones that induced sAHP in the work of Tzingounis et al. [19]. If channels underlying sAHP are hippocalcin targets then the observed HPCA-YFP translocations within a dendritic tree could indicate their possible clustered distribution within the plasma membrane.

Thus, our cellular studies confirm that biophysical properties of hippocalcin may support its presumable physiological roles in the hippocampus and suggest that it is the very sensitive tool for decoding complex spatio-temporal changes of [Ca^2+^]_i_ taking place in processes of hippocampal neurons into site-specific translocations of this Ca^2+^ sensor to its membrane-anchored targets.

## Figures and Tables

**Fig. 1:**
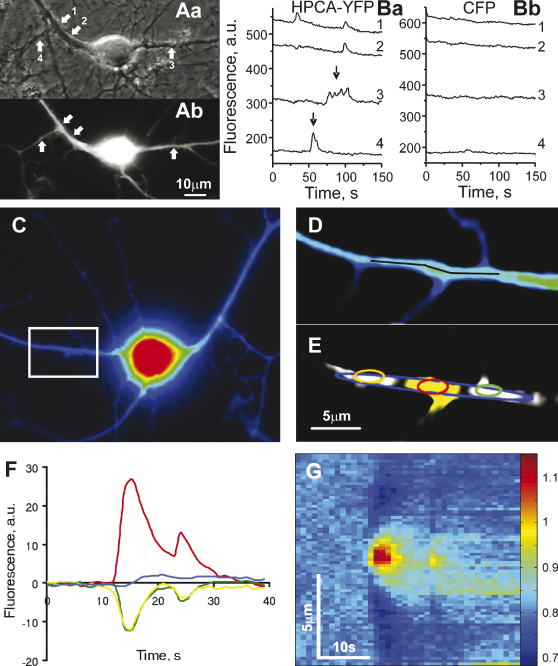
HPCA-YFP spontaneously translocates to certain sites in spatially restricted domains of neuronal processes. (A) Phase contrast ‘a’ and fluorescent ‘b’ images of a hippocampal neuron (11 DIV) growing in a low-density primary hippocampal culture. This neuron was co-transfected with plasmids encoding hippocalcin tagged with a yellow fluorescent protein, HPCA-YFP, and a cyan fluorescent protein, CFP. Arrows indicate regions of interest, ROIs, from which time course of fluorescence changes was recorded and shown in B. The fluorescent image in Ab was taken for YFP fluorescence settings. (B) Traces in a represent changes in HPCA-YFP fluorescence whereas ones in b represent CFP fluorescence changes observed simultaneously. Numbers by the traces correspond to ones of arrows in (Aa). Arrows in (Ba) indicate one and multiple peak transient increases of HPCA-YFP fluorescence. (C) HPCA-YFP fluorescent image of a neuron (10 DIV), in which a spontaneous HPCA-YFP translocation was revealed in a part of dendrite indicated by a box. (D) A higher magnification image of a dendritic branch shown in a boxed area in (C). A time course of changes in HPCA-YFP fluorescence along a black curve within the dendrite is shown in (G). (E) A pseudo-colored image of the dendritic branch taken at a maximum of spontaneous HPCA-YFP translocation. A decrease in HPCA-YFP fluorescence is shown in white whereas an increase is shown in yellow; there were no changes in the fluorescence in black areas. HPCA-YFP translocation was directed to a certain site in the dendrite of about 5 μm with the protein collected from a region of about 20 μm. Simultaneously, no translocations were observed in the same and other dendritic branches of the same neuron. Time courses of HPCA-YFP fluorescence changes in colored ROIs depicted in E are shown in (F). (F) An increase of HPCA-YFP fluorescence in the site of translocation (red trace) was accompanied by a decrease in neighboring sites (green and yellow traces) preserving a total amount of HPCA-YFP (blue trace) in the dendritic compartment. (G) A spatio-temporal pattern of changes in HPCA-YFP fluorescence during a translocation event observed along the dendritic compartment. A color map represents relative changes of HPCA-YFP fluorescence against CFP background. Similar results were obtained for other analyzed spontaneous HPCA-YFP translocations (30 events observed in 9 neurons).

**Fig. 2:**
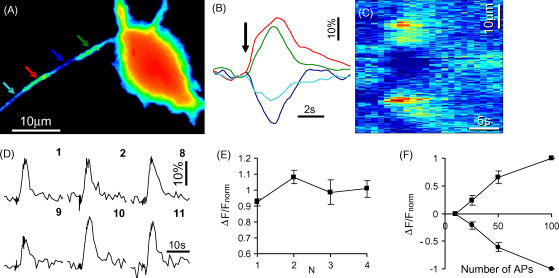
Action potentials evoke fast, synchronous, reversible, site-specific and Ca^2+^-dependent HPCA-YFP translocations in neuronal processes. (A) HPCA-YFP fluorescent image of a 10 DIV hippocampal neuron taken at the maximum of AP-induced HPCA-YFP translocation. Colored arrows indicate ROIs from which relative fluorescence changes were measured during stimulation and shown in (B). Trace colors in (B) match arrow colors in (A). An onset of stimulation (30 stimuli at 30 Hz) leading to synchronous HPCA-YFP translocations to 2 closely located sites is indicated by an arrow. (C) A spatio-temporal pattern of changes in HPCA-YFP fluorescence during the AP-induced translocation event. Changes in HPCA-YFP fluorescence were measured along a curve drawn within the process (not shown) from its beginning up to a cyan arrow in (A). (D) A time course of changes in HPCA-YFP fluorescence during translocations induced by sequential APs-stimulations. The neuron (9 DIV) was in a voltage clamp mode (Whole-cell configuration) and was stimulated with 2 ms depolarization to 0 mV (20 times at 30 Hz). Episodes of stimulation were repeated 14 times with 2 min intervals. Numbers indicate a translocation episode number. (E) Normalized amplitudes of HPCA-YFP translocations to four equal consequently delivered AP stimulations (50 APs at 20 Hz; *n* = 45 ROIs from 5 neurons). (F) The dependence of amount of translocated HPCA-YFP on the number of APs in a train. Neurons expressing HPCA-YFP were stimulated in a current clamp mode to generate APs (20 Hz; 2 ms current stimuli) 10, 25, 50 and 100 times. Episodes of stimulation (repeated each 2 min) resulted in HPCA-YFP translocation to certain sites. Amplitudes of HPCA-YFP fluorescence changes in ROIs placed over these sites and over immediately adjacent sites (where HPCA-YFP fluorescence was decreased) were calculated, normalized (to translocation amplitudes in response to 100 APs) and depicted in the graph as a function of number of APs in a train (*n* = 59 ROIs from 6 neurons).
