# Supplementary material for: Hippocalcin signaling via site-specific translocation in hippocampal neurons
Source: Neurosci Lett. 2008 Sep 12;442(2):152–7. doi: 10.1016/j.neulet.2008.06.089 (PMC2572729; doi:10.1016/j.neulet.2008.06.089)
Supplement: Supplementary file 1 [file mmc1.doc]

Figure A. Ca^2+^ dependence of HPCA-YFP translocation

**A-C**. Neurons expressing HPCA-YFP and loaded with fura-2 were stimulated in perforated whole cell configuration to activate voltage-operated calcium channels (6 sec, 16 Hz; depolarization from -60 to 0 mV for 20 msec). The stimulations were applied in a normal (2.0 mM) and low (0.5 mM) Ca^2+^ extracellular solutions. HPCA-YFP and fura-2 fluorescence were monitored in sites to which HPCA-YFP translocated. A stimulation episode in a normal extracellular solution (**A**) induced [Ca^2+^]_i_ increase and HPCA-YFP translocation that was practically absent in the solution with low level of Ca^2+^ (0.5 mM) although some increase in [Ca^2+^]_i_ was still present (**B**) as a result of stimulation. Translocation was resumed after Ca^2+^ wash-in (**C**). HPCA-YFP translocation (black traces) followed [Ca^2+^]_i_ elevation (grey traces) with a delay of 1-2 sec (**A**). **D**. A summary graph showing a significant decrease (p<0.05) in amplitudes of translocation in the low Ca^2+^solution as compared to the normal one (n=32 ROIs from 5 neurons). **E**. A histogram of relative changes of HPCA-YFP and HPCA (E85,121Q)-CFP fluorescence calculated at the peaks of increases of HPCA fluorescence in sites of translocation (n=38 ROIs recorded in 5 neurons; p<0.01). HPCA (E85,121Q)-CFP is a hippocalcin mutant designed to prevent Ca^2+^ binding to its EF-hands 2 and 3. Translocation was induced when [Ca^2+^]_i_ was elevated by trains of APs.
